# First-Principles Thermodynamics of Hydrogen Absorption in Binary C15 Laves Phases

**DOI:** 10.1021/acs.chemmater.5c01925

**Published:** 2026-01-07

**Authors:** Claire A. Paetsch, Anirudh Raju Natarajan

**Affiliations:** † Laboratory of Materials Design and Simulation (MADES), Institute of Materials, 27218 École Polytechnique Fédérale de Lausanne, CH-1015 Lausanne, Switzerland; ‡ National Centre for Computational Design and Discovery of Novel Materials (MARVEL), 27218 École Polytechnique Fédérale de Lausanne, CH-1015 Lausanne, Switzerland

## Abstract

Intermetallic compounds are attractive candidates for hydrogen storage applications. This study investigates the thermodynamics of hydrogen absorption in binary AB_2_ Laves phases with the C15 crystal structure. First-principles calculations, cluster expansion models, and statistical mechanics simulations are employed to determine the pressure–composition isotherms for two prototypical Laves phases: ZrMo_2_ and ZrV_2_. Our calculations show that ZrMo_2_ accommodates hydrogen exclusively within A_2_B_2_ coordinated tetrahedral sites. In contrast, ZrV_2_ accommodates hydrogen over both A_2_B_2_ and AB_3_ coordinated tetrahedral sites. Finite-temperature simulations reveal that hydrogen atoms can occupy neighboring edge-sharing tetrahedra and are separated by a distance close to the Switendick criterion in ZrV_2_. The occupation of both interstitial site types increases the hydrogen storage capacity of ZrV_2_ as compared to ZrMo_2_. Building on this insight, we perform a high-throughput search of binary C15 Laves phases and identify several promising candidates that can accommodate hydrogen across multiple interstitial sites. The results of this study provide chemical guidelines for tuning the hydrogen storage capacity of intermetallic compounds.

## Graphical abstract

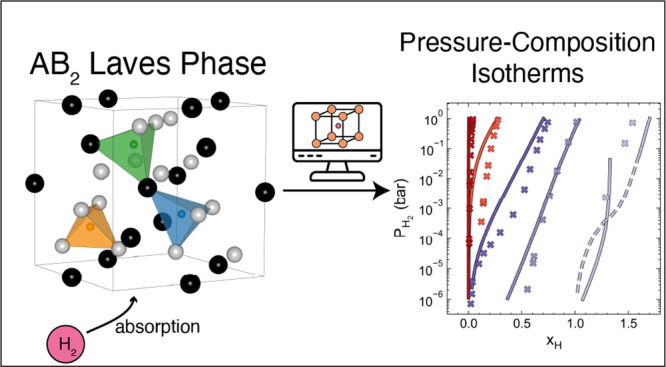

## Introduction

Hydrogen is envisioned to play a major role in decarbonizing the energy economy. However, its low volumetric density presents a major challenge. Storing hydrogen in solid-state materials offers a path toward increasing its energy density and improving safety.^1–5^ Intermetallic compounds are a promising class of solid-state hydrogen storage materials.^3–5^ Attaining theoretical capacities for hydrogen storage in intermetallic compounds is often challenging due to unfavorable thermodynamics and slow kinetics. An incomplete understanding of relationships between chemistry, crystal structure and hydrogen uptake limits our ability to design better hydrogen storage materials.

Laves phases are a particularly interesting and common class of intermetallic compounds.^6–8^ Single-phase Laves intermetallics with a wide range of compositions have been successfully synthesized through conventional techniques. Described by the prototypical formula AB_2_, these compounds are formed by combining a larger element A with a smaller element B. The crystal structure of a Laves phase can be viewed as a sequence of stacked kagome and triangular layers. While infinite stacking sequences are possible, structure prototypes referred to as C15, C14, and C36 are commonly observed in experiments.^7,9^ Previous studies discuss the structural characteristics and properties of the three Laves phases.^6,9,10^

Several studies have investigated the absorption of hydrogen within Laves phases with experiments.^11^ Pressure–composition isotherms coupled with advanced characterization techniques have proven invaluable in elucidating links between intermetallic chemistry and hydrogen storage capacity. For instance, a recent study of the ZrV_2_ Laves phase found that at high hydrogen compositions, the distance between neighboring hydrogen atoms may fall below 2.1 Å,^12^ violating the well-known Switendick criterion for minimum H–H separation in a stable hydride.^13^ More recent work has focused on exploring “high-entropy” or multicomponent Laves phases to improve storage capacities and tune absorption/desorption partial pressures.^14–18^ Despite these advances, a fundamental understanding of atomistic mechanisms of hydrogen storage and the effects of temperature and chemistry on storage capacities in Laves phase compounds remains elusive.

Hydrogen dissolved in an intermetallic compound occupies well-defined interstitial sites within the host crystal structure. In Laves phase compounds, hydrogen is known^19^ to occupy three distinct types of tetrahedral sites as illustrated in Figure 1. The coordination environment of each interstitial site is comprised of four metal atoms. As shown in Figure 1, the tetrahedron depicted in green contains two A and two B atoms, the site coordinated by three B and one A is highlighted in blue, and the third interstitial site, shown in orange, is coordinated by four B atoms. These three interstitial sites are typically referred to by their local metal coordination as A_2_B_2_, AB_3_, and B_4_, respectively. Of the three possible coordination environments, experiments indicate that the A_2_B_2_ sites are usually preferred across intermetallic chemistries, with some compounds also accommodating hydrogen atoms within the AB_3_ sites.^20,21^

**Figure 1:**
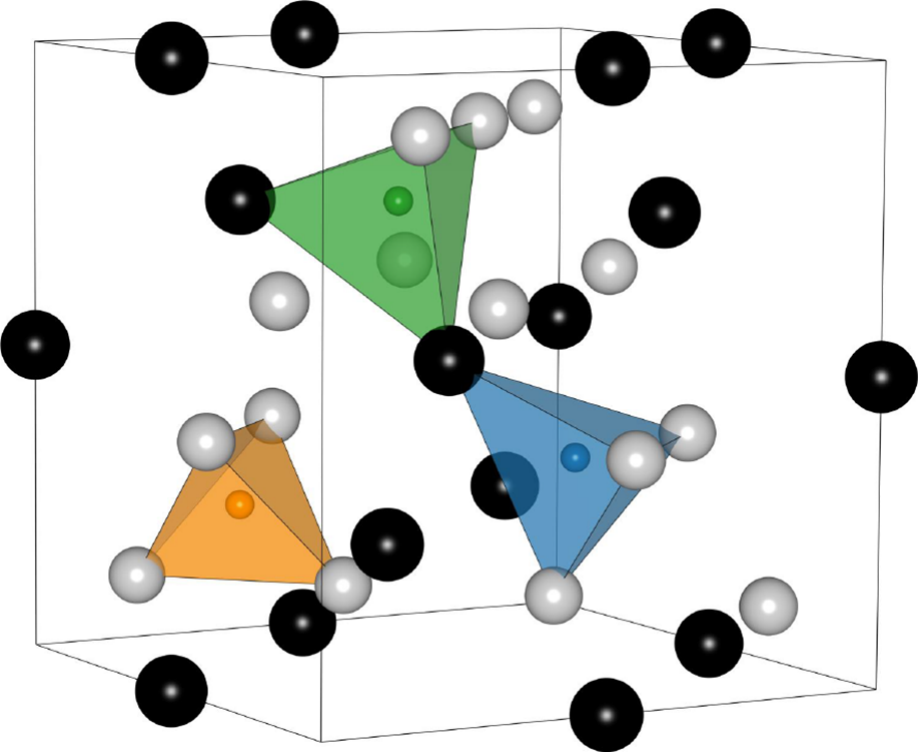
Crystal structure of C15 Laves phase (AB_2_) with element A (black) and element B (light gray), highlighting hydrogen interstitial sites by coordination: A_2_B_2_ (green), AB_3_ (blue), and B_4_ (orange).

Laves phases can theoretically accommodate up to 17 hydrogen atoms per formula unit (AB_2_) of the compound. Of the 17 theoretically available interstitial sites, 12 have the A_2_B_2_ coordination, 4 are within a AB_3_ tetrahedron and only one site is coordinated by 4 B atoms. However, the maximum hydrogen concentrations in experiment rarely exceed five hydrogen per formula unit.^20,22–24^ Repulsive interactions between hydrogen atoms limit the set of interstitial sites that can be simultaneously occupied within the host intermetallic compound. Restricting the distances between hydrogen atoms to 2.1 Å, and disallowing face-sharing tetrahedra from being simultaneously occupied by hydrogen reduces the storage capacity of Laves phases to 6 and 6 $\frac{1}{3}$ per formula unit in the C15 and C14 structures.^19^

The theoretical capacity proposed by Shoemaker and Shoemaker^19^ is seldom achieved in experiments, likely due to thermodynamic preferences for site occupancies. Figure 2 illustrates the effects of constraining both the minimum distance between pairs of hydrogen atoms and the tetrahedral coordination environments that are available for hydrogen absorption. Figure 2 plots the theoretical maximum hydrogen capacity as a function of the minimum allowed hydrogen–hydrogen distance (*d*_H–H_^min^), scaled by the lattice parameter (*a*) of the C15 Laves phase. This scaling allows for a direct comparison of storage capacities across Laves phases with different chemistries and lattice parameters. The maximum capacity is achieved when all H–H pairs can be populated (*d*_H–H_^min^/*a* = 0). The green curve shows that when hydrogen exclusively occupies the A_2_B_2_ tetrahedra, the maximum composition decreases to 4 hydrogen atoms per metal atom, which is equivalent to 12 hydrogen atoms per formula unit. Figure 2 also illustrates the range of hydrogen pair distances below which the Switendick criteria would be violated in intermetallic binary C15 Laves phases. The maximum theoretical storage capacities in experimentally synthesizable C15 phases are expected to range between ≈0.6–1.6. Compounds with larger lattice parameters will result in higher storage capacities, while smaller lattice parameters will significantly reduce theoretical capacities. The storage capacity when hydrogen dissolves into the AB_3_ site (blue curve in Figure 2) is lower than the A_2_B_2_ sites. We do not consider the B_4_ tetrahedron in Figure 2 due to the small number and high absorption energies of these sites.

**Figure 2:**
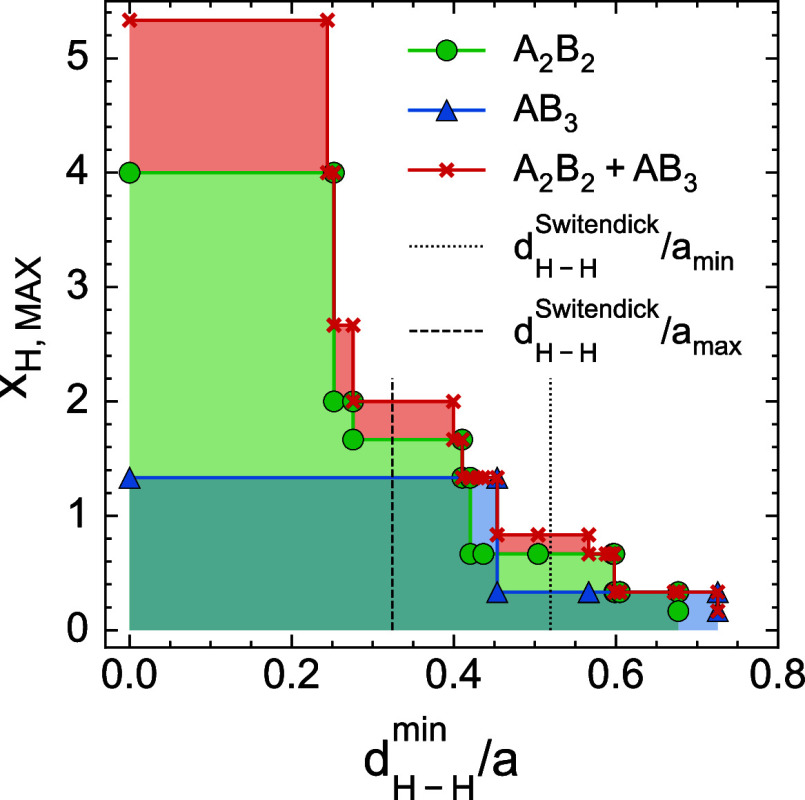
Maximum achievable hydrogen compositions as a function of the minimum H–H pair distance (*d*_H–H_^min^) normalized by the lattice parameter (*a*) of the C15 Laves phase. The curves correspond to maximum hydrogen occupancy in A_2_B_2_ sites (green), AB_3_ sites (blue), and dual-site occupancy (red). Vertical dotted lines represent theoretical lower and upper bounds on capacity based on the Switendick criterion applied to the smallest (*a*_min_) and largest computed lattice constants (*a*_max_) for binary C15 Laves phases.

Although Laves phases with larger lattice parameters can theoretically store more hydrogen per formula unit, their reduced volumetric storage capacities make them unsuitable for stationary applications. Figure 2 indicates that storage capacities can be increased by unlocking multiple interstitial sites for hydrogen occupation. Allowing for hydrogen absorption into both AB_3_ and A_2_B_2_ interstitial sites improves storage capacities to range between 0.8–2.0 hydrogen per metal atom. While Figure 2 provides a guide toward choosing Laves phase compounds with higher storage capacities, in practice, several factors play a significant role toward enabling the commercial viability of these intermetallics. For example, the bonding between hydrogen and the metal determines absorption and desorption pressures, as well as the interstitial sites that can be occupied by hydrogen. Factors such as kinetics will ultimately set the charge and discharge rates for hydrogen storage and release. Rigorous atomistic calculations are required to elucidate the precise relationships between chemical bonding, the available interstitial sites in a Laves phase, and the partial pressures of hydrogen required to store or release hydrogen from intermetallic compounds at finite-temperature.

In this study, we employ first-principles calculations, cluster expansion based atomistic surrogate models, finite-temperature statistical mechanics, and high-throughput DFT calculations to systematically investigate the interactions between hydrogen and binary intermetallic C15 Laves phases. We begin by computing pressure–composition isotherms (PCT) for the prototypical ZrMo_2_ and ZrV_2_ Laves phases. First-principles calculations reveal distinct energetic preferences for hydrogen dissolution into the two Laves phases. While ZrMo_2_ only accommodates H in the A_2_B_2_ tetrahedral site, ZrV_2_ has roughly similar energetic preferences for hydrogen uptake into both the A_2_B_2_ and AB_3_ tetrahedral site. Hydrogen binds strongly to ZrV_2_, while there is significantly less binding between hydrogen and the metal atoms in ZrMo_2_. The differences in site energetics and binding energies result in drastically different behavior with regard to hydrogen storage capacities and PCT curves. ZrV_2_ allows for significantly higher hydrogen uptake than ZrMo_2_ at similar hydrogen partial pressures at elevated temperatures. Our rigorous first-principles calculations and statistical mechanics simulations suggest that the higher capacity of ZrV_2_ is due to the similar energetic preferences for hydrogen site occupancies. Inspired by this, we search across all binary Laves phases for viable candidates that may enable hydrogen uptake across multiple tetrahedral interstitial sites.

## Methods

First-principles calculations are performed using the Vienna Ab-initio Simulation Package (VASP)^25–27^ with the projector-augmented wave (PAW) method and Perdew–Burke–Ernzerhof (PBE) functionals.^28^ Spin polarization is included for systems containing magnetic elements such as V, Cr, Fe, Co, Ni, and Mn. All calculations employ a plane-wave cutoff energy of 500 eV and a *k*-point mesh of 55 Å corresponding to a 13 × 13 × 13 mesh for the primitive cell of the Laves phase. Atomic positions, cell shape, and volume are fully relaxed until forces are below 0.01 eV/Å.

Finite-temperature pressure–composition isotherms are computed through rigorous statistical mechanics techniques informed by an atomistic surrogate model parametrized on a training data set of first-principles calculations. When an intermetallic compound, α, is in equilibrium with an environment at a temperature *T* and a partial pressure of hydrogen *p*_H_2__, thermodynamic equilibrium dictates that

$$\mu_{H,\alpha }(T,x_{H})=\frac{1}{2}\mu_{H_{2}}(T,p_{H_{2}})$$ 1

where μ_H,α_(*T*, *x*_H_) is the chemical potential of hydrogen in the intermetallic compound with a hydrogen concentration $x_{H}=\frac{N_{H}}{N_{M}}$ at a temperature *T*. The hydrogen concentration is normalized relative to the number of metal atoms. The thermodynamic equilibrium condition can be used together with additional assumptions on the sources of entropy within the intermetallic compound to relate the chemical potential of hydrogen within the α phase to the equilibrium pressure of hydrogen in the environment:

$$p_{H_{2}}=p_{H_{2}}^{0}\exp\left(2\frac{\mu_{H,\alpha }^{\mathrm{config}}(T,x_{H})+\Delta g_{H}^{\mathrm{vibrational}}(T,p_{H_{2}}^{0})}{k_{B}T}\right)$$ 2

where $\mu_{H,\alpha }^{\mathrm{config}}={\left(\frac{\partial g_{\alpha }^{f,\mathrm{config}}}{\partial x_{H}}\right)}_{T,N_{M}}$ is the chemical potential of hydrogen in the intermetallic compound, and Δ*g*_H_^vibrational^(*T*, *p*_H_2__^0^) corresponds to the variations in the free energy due to the vibration of hydrogen atoms. The assumptions used in deriving eq 2 are detailed in Section S1. As outlined in Section S1, we assume that the primary source of entropy in the intermetallic compound arises from the various ways in which hydrogen can be distributed over the interstitial sites of the metallic host. Equation 2 also accounts for vibrational entropy arising from zero-point vibrational motion of hydrogen due to its low mass. Other sources of entropy, such as the vibrational entropy of the intermetallic compound, are neglected.

The relationship between the hydrogen chemical potential in the intermetallic compound and the equilibrium composition of hydrogen is the primary input to eq 2 required to compute pressure–composition isotherms. Statistical mechanics techniques can be employed to compute μ_H,α_^config^(*x*_H_, *T*). Within the grand-canonical ensemble, the ensemble average of the hydrogen composition within the intermetallic compound is related to the chemical potential as

$$x_{H}(T,\mu_{H,\alpha }^{\mathrm{config}})=\frac{1}{Z}\underset{\overset{\to }{\sigma }}{\sum }\frac{N_{H,\overset{\to }{\sigma }}}{N_{M}}\exp\left(-\frac{E_{f}(\overset{\to }{\sigma })-\mu_{H,\alpha }^{\mathrm{config}}N_{H,\overset{\to }{\sigma }}}{k_{B}T}\right)$$ 3

where $E_{f}\left(\overset{\to }{\sigma }\right)$ is the formation energy of an arrangement (denoted $\overset{\to }{\sigma }$ ) of $N_{H}\left(\overset{\to }{\sigma }\right)$ hydrogen atoms over the interstitial sites of *N*_M_ metal atoms, and *Z* is the partition function. Monte Carlo simulations within the semigrand canonical ensemble can be employed to approximate eq 3, provided the formation energies of arbitrary hydrogen arrangements can be rapidly computed.

On-lattice cluster expansions^29–31^ are ideal surrogate models to capture the ordering energetics of hydrogen and vacancies over the network of interstitial sites within a metal host structure. Cluster expansion models have been extensively used in elucidating the effects of interstitial impurities such as carbon, nitrogen, hydrogen and oxygen dissolved in pure metals and alloys.^32–37^ Within the cluster expansion formalism, each interstitial site, *i*, is either occupied by a hydrogen atom or a vacancy. Occupation variables, σ_*i*_, are assigned to each site and take a value of zero if the site is vacant or a value of one if the site is occupied by hydrogen. Any arbitrary arrangement of hydrogen and vacancy can then be described by the collection of occupation variables $\vec{\sigma }=[\sigma_{1},\sigma_{2},\cdot \cdot \cdot ]$ . The energy of an arbitrary arrangement of hydrogen and vacancies within the interstitial network is given by^29,30^

$$E_{f}(\overset{\to }{\sigma })=V_{0}+\underset{i}{\sum }V_{i}\sigma_{i}+\underset{ij}{\sum }V_{ij}\sigma_{i}\sigma_{j}+\underset{ijk}{\sum }V_{ijk}\sigma_{i}\sigma_{j}\sigma_{k}+\cdot \cdot \cdot$$ 4

where *V*_0_, *V*_*i*_, *V*_*ij*_, *V*_*ijk*_, ···, also known as effective cluster interactions (ECI), are expansion coefficients that can be related to the energies of the empty intermetallic host, the energy contributions of noninteracting hydrogen atoms within the host compound, the pair interactions between hydrogen atoms dissolved within the host and so on. The indices *i*, *j*, *k* refer to different interstitial sites within the host crystal. The ECI’s are learned from a small pool of electronic structure calculations containing symmetrically distinct arrangements of hydrogen and vacancies within binary Laves phases. On-lattice cluster expansions are parametrized with the *Clusters Approach to Statistical Mechanics* (CASM) code.^38,39^ The cluster expansion models are then used with semigrand canonical Monte Carlo simulations to approximate eq 3.

## Results

We begin our analysis by investigating the thermodynamics of hydrogen absorption into C15 ZrMo_2_ and ZrV_2_ Laves phases. Both intermetallic compounds have been extensively studied with experiment. The investigation by Pebler^22^ measured pressure–composition isotherms in both compounds across temperatures ranging from 0–700 °C. Other researchers have measured the occupancies of various tetrahedral interstitial sites in the C15 Laves phases, as well as hydrogen distributions in these two Laves phases.^12,23,24,40,41^

Figure 3 presents the zero-Kelvin formation energies of hydrogen-vacancy orderings within the A_2_B_2_ and AB_3_ tetrahedra of the C15 ZrMo_2_ and ZrV_2_ Laves phases. Formation energies in Figure 3 are calculated relative to the pure binary Laves phase and an isolated hydrogen molecule. For the ZrMo_2_ calculations, hydrogen was constrained to occupy either A_2_B_2_ or AB_3_ sites, whereas for ZrV_2_, simultaneous occupation of both site types was permitted.

**Figure 3:**
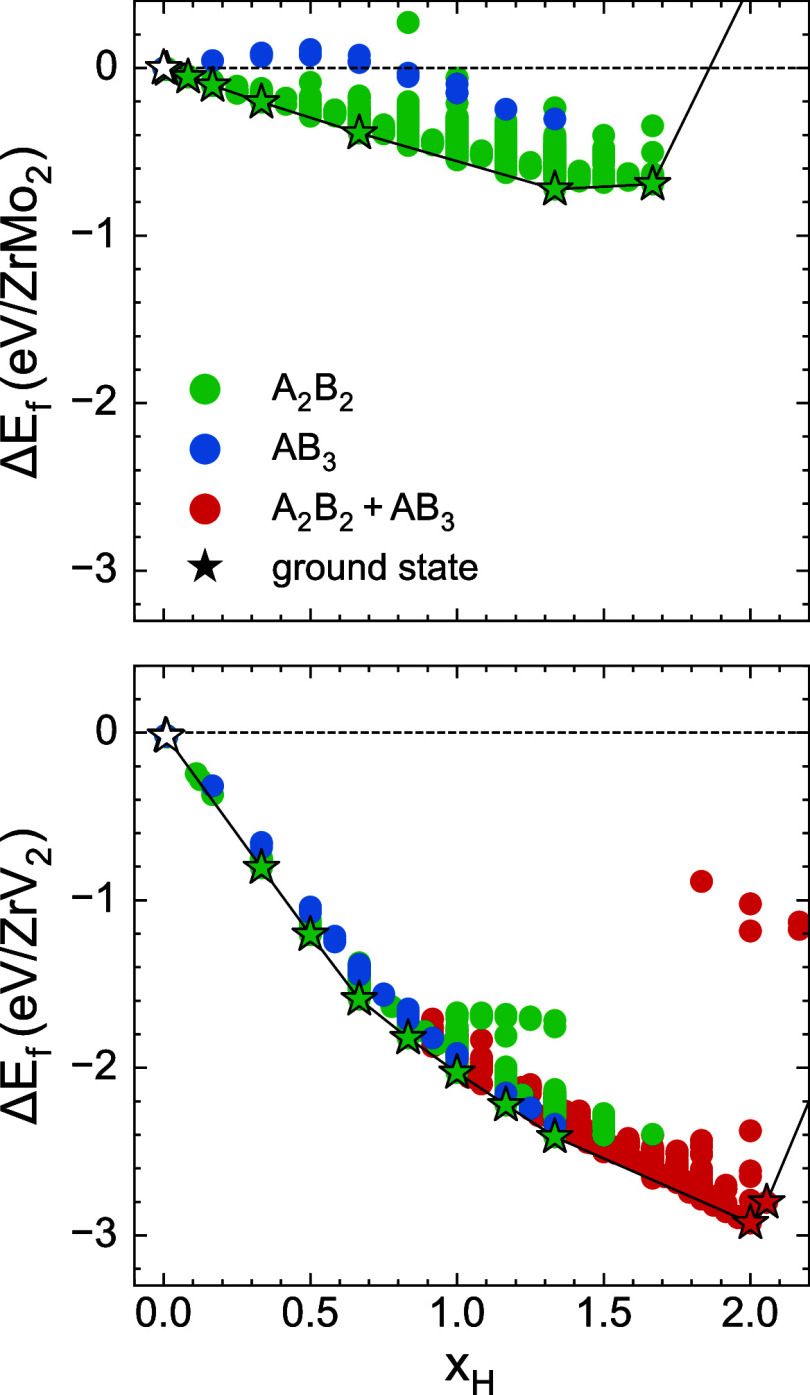
DFT formation energies for hydrogen-vacancy orderings in ZrMo_2_ (top) and ZrV_2_ (bottom). Each ordering is colored based on the set of interstitial sites that are occupied. Green points contain hydrogen atoms distributed over A_2_B_2_ sites, blue corresponds to AB_3_ sites, and red corresponds to H atoms occupying both tetrahedral sites. The 0 K convex hull and ground states are shown by the black line and stars, respectively.

The formation energies of Figure 3 reveal very different site preferences for hydrogen between the two Laves compounds. In ZrMo_2_, orderings with hydrogen in AB_3_ tetrahedra have higher formation energies than those with hydrogen occupying only A_2_B_2_ tetrahedra. The significant difference in formation energies suggests that AB_3_ sites are unlikely to be populated in this compound, even at elevated temperatures. In contrast, both interstitial sites are energetically competitive in C15 ZrV_2_. Although all zero-Kelvin ground states up to a composition of *x*_H_ = 1.33 accommodate hydrogen within the A_2_B_2_ sites, the energetic proximity of mixed-site configurations (containing H in both A_2_B_2_ and AB_3_ tetrahedra, shown in red in Figure 3) to the convex hull suggests that AB_3_ sites will be populated at finite temperatures. The formation energies also indicate that hydrogen binds more strongly to ZrV_2_ than to ZrMo_2_. For example, when referenced to an isolated H_2_ molecule, the formation energy of the ground state ordering with a composition of $x_{H}=\frac{4}{3}$ is −2.41 eV/formula unit in ZrV_2_ and −0.72 eV/formula unit in ZrMo_2_.

Despite the qualitative differences in site occupancies and hydrogen dissolution energies between ZrV_2_ and ZrMo_2_, both intermetallic compounds form similar low-temperature ordered structures. At a composition of $x_{H}=\frac{4}{3}$ , the stable phase adopts the crystal structure shown in Figure 4. The ordering is characterized by hydrogen atoms arranged in a helical pattern along the [001] direction of the parent C15 Laves phase. As illustrated in the inset of Figure 4, these helices consist of channels where hydrogen atoms and vacancies alternate within adjacent A_2_B_2_ tetrahedral sites. The alternating arrangement is necessary to avoid the large repulsive interaction that would arise from filling face-sharing tetrahedra. The resulting structure maximizes the H–H separation while maintaining a high packing density within the interstitial channel. The ground state ordering with a composition of $x_{H}=\frac{4}{3}$ belongs to the *I*4_1_/*a* space group, which has been previously reported to be observed in a deuterated ZrV_2_D_3.6_ ^42^ and for the HfV_2_ ^43^ intermetallic compound at low temperatures.

**Figure 4:**
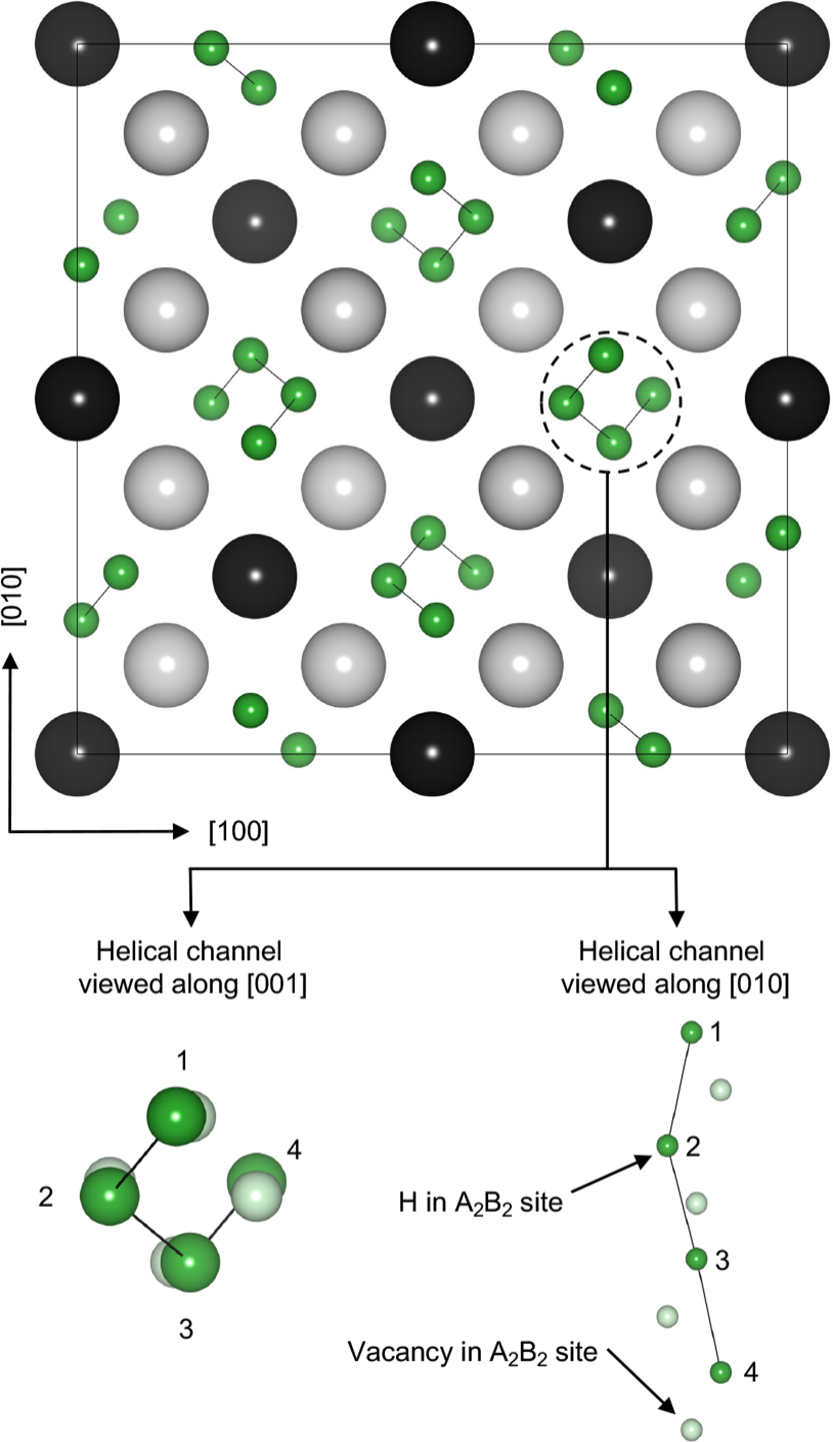
Zero-Kelvin ground state ordering with a composition of $x_{H}=\frac{4}{3}$ . The larger element, A is shown in black; the smaller element B is in light gray. Hydrogen atoms are drawn in dark green. The inset (shown below) also illustrates the vacant sites within a single channel of A_2_B_2_ sites in light green.

In the composition range $\frac{2}{3}\leq x_{H}\leq \frac{4}{3}$ , stable phases in both compounds exhibit hydrogen-vacancy arrangements that closely resemble the structure shown in Figure 4. The helical [001] channels of hydrogen are progressively filled with increasing hydrogen composition. At lower hydrogen compositions of $x_{H}=\frac{2}{3}$ , the [001] channels are half-filled relative to the $x_{H}=\frac{4}{3}$ structure. As hydrogen composition increases, the remaining sites within the channels are occupied until the arrangement of Figure 4 is attained. While both Laves phases generally follow this trend, the ZrV_2_ system presents an exception. This Laves phase contains a single ground state ordering that involves the occupation of an A_2_B_2_ site located outside of the primary [001] channels.

Figure 5 presents the minimum hydrogen–hydrogen (H–H) distances for the zero-Kelvin ground states in both C15 Laves phases. The dashed line at 2.1 Å represents the minimum separation limit established by the Switendick criterion. The calculated ground states largely adhere to this criterion. For compositions *x*_H_ < 0.5, all H–H distances exceed 2.1 Å. At higher hydrogen compositions, most ground states have at least one H–H pair distance that approaches the Switendick limit. A few ground states in ZrV_2_ are predicted to have a minimum H–H distance marginally below the 2.1 Å threshold. However, this deviation is likely within the numerical accuracy of the DFT calculations and does not constitute a clear violation of the Switendick criterion.

**Figure 5:**
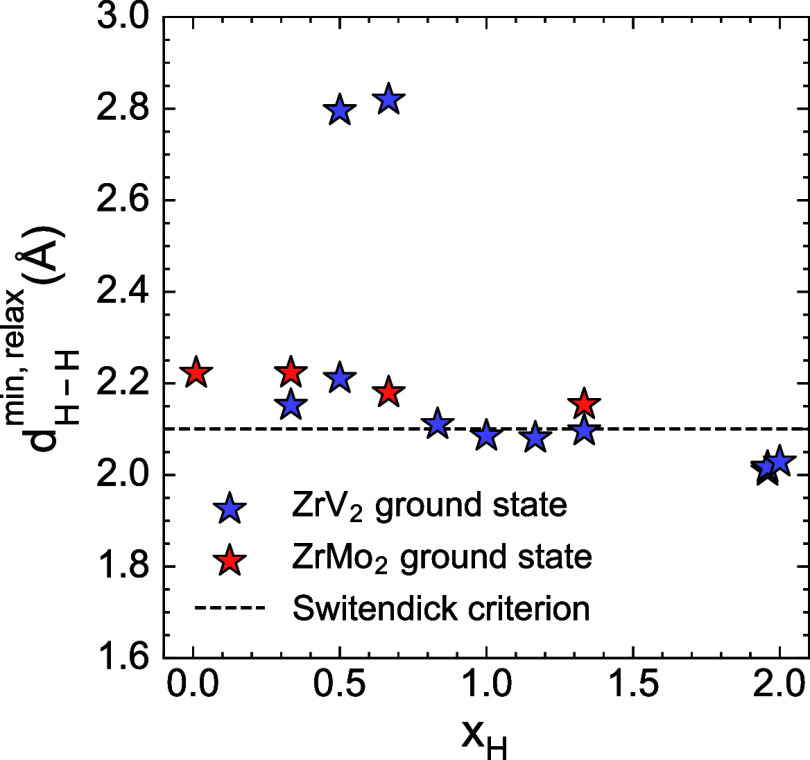
Minimum distances between hydrogen pairs in the zero-Kelvin ground states of ZrMo_2_ and ZrV_2_.

We parametrized two cluster expansion (CE) models to study hydrogen absorption in ZrMo_2_ and ZrV_2_ using the CASM software package. Both models consider hydrogen-vacancy ordering over the tetrahedral interstitial sites of the C15 Laves phase. Disorder over the metallic sites was not included as the Laves phases are expected to be intolerant of defects. The CE model for ZrMo_2_ was restricted to the A_2_B_2_ sublattice as the formation energies in Figure 3 indicate that the energies of hydrogen in AB_3_ sites are significantly higher than those in A_2_B_2_ sites. The CE model for ZrV_2_ accounted for hydrogen ordering over both A_2_B_2_ and AB_3_ interstitial sublattices and allowed for cluster interactions that spanned both site types.

The CE models for ZrMo_2_ and ZrV_2_ were trained on DFT formation energies of 990 and 771 symmetrically distinct hydrogen-vacancy arrangements, respectively. These training sets were constructed iteratively. An initial CE was fit to a small set of DFT calculations with structures containing up to 30 metal atoms. This CE was then used in Monte Carlo simulations to predict new low-energy ordered structures. The formation energies of low-energy orderings were subsequently calculated with DFT and added to the training set. The process was repeated until no new ground states were found. All DFT relaxed structures were mapped onto the ideal parent Laves structure using a structure matching algorithm.^44^ We retained only configurations with an atomic deformation cost below 0.04, calculated using the algorithm of Thomas et al.^44^ The training data also included a large cell containing 64 formula units of the Laves phase to correctly estimate pair interactions between H atoms at varying distances. The energies of these dilute pair defects were obtained by relaxing only atomic positions. We used LASSO regression to parametrize the CE and prevent overfitting. The regularization parameter for the LASSO fit was determined using 12-fold cross-validation. Uniform weights were applied to all configurations in the training set, except for the 0 K ground states, which were given a higher weight.

Figure 6 compares the formation energies predicted by CE models with those calculated by DFT for hydrogen-vacancy orderings in the ZrMo_2_ and ZrV_2_ C15 Laves phases. The models demonstrate good agreement with the DFT data, achieving root-mean-squared errors (RMSE) of 5.6 and 13.8 meV/metal atom for the ZrMo_2_ and ZrV_2_ systems, respectively. Both CEs accurately reproduce the important ground states at zero Kelvin. Some DFT orderings such as the ground state with a composition of *x*_H_ = 0.5 in ZrV_2_ are not captured. However, this is unlikely to affect finite-temperature predictions as this ordering is stable within a very narrow range of hydrogen chemical potentials. The CE models also predict some additional ground states not present in the DFT training sets. The DFT formation energies of these orderings are within 4–10 meV/atom of the DFT convex hull. The CEs were carefully tested to ensure that important ground states are reproduced up to a composition of *x*_H_ = 4/3 and *x*_H_ = 2 in the ZrMo_2_ and ZrV_2_ systems.

**Figure 6:**
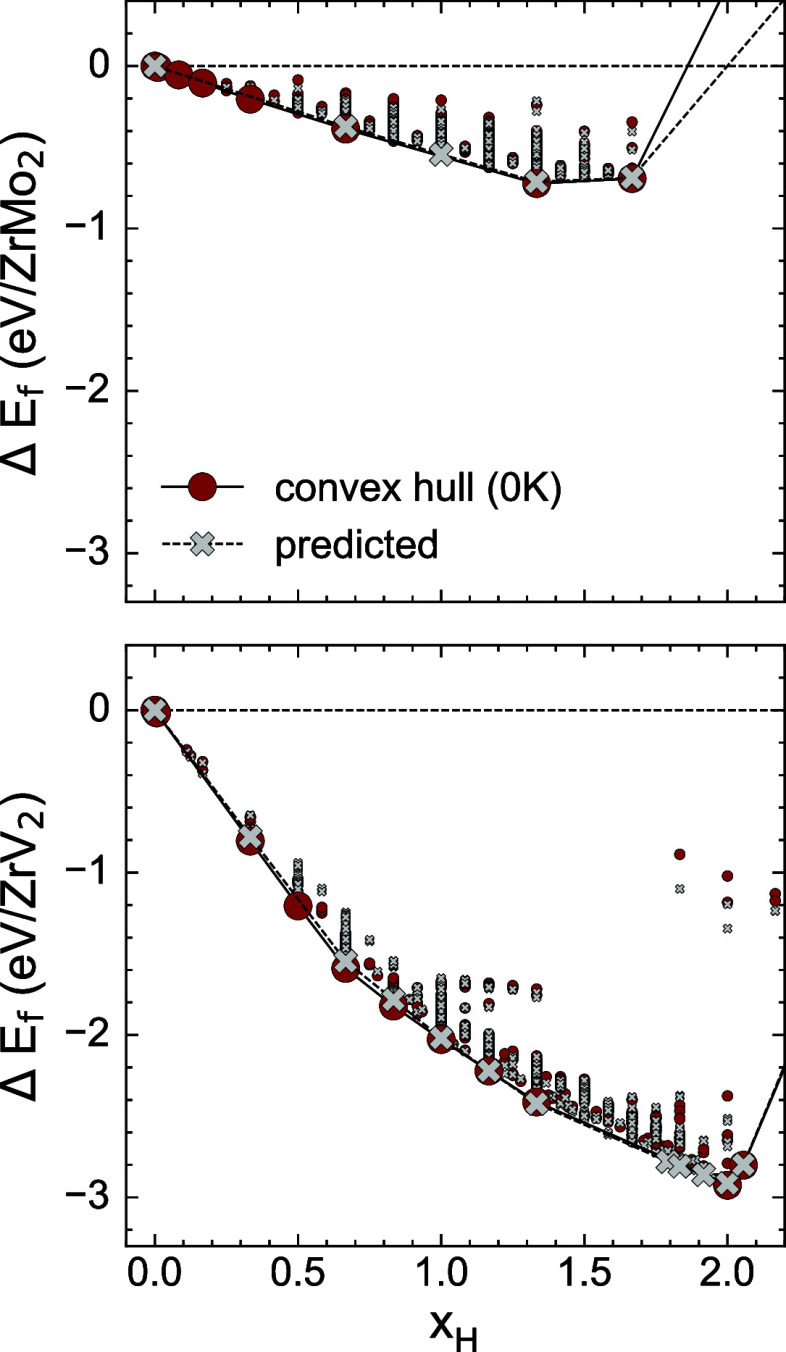
DFT (red) and cluster-expanded (gray) formation energies of hydrogen-vacancy orderings within the ZrMo_2_ (top) and ZrV_2_ (bottom) C15 Laves phases. Ground state orderings are shown by larger markers. DFT convex hulls are shown by the solid line, while the CE convex hull is denoted by the dashed line.

Figure 7 compares the pressure–composition isotherms computed from our CE models with the experimental measurements of Pebler and Gulbransen.^22^ Finite-temperature Monte Carlo simulations are employed to estimate ensemble averages of the hydrogen compositions at fixed chemical potential and temperature. The hydrogen chemical potential is then related to the hydrogen pressure through eq 2. Overall, the computational predictions of the pressure–composition isotherms for both systems are in excellent agreement with experimental measurements.

**Figure 7:**
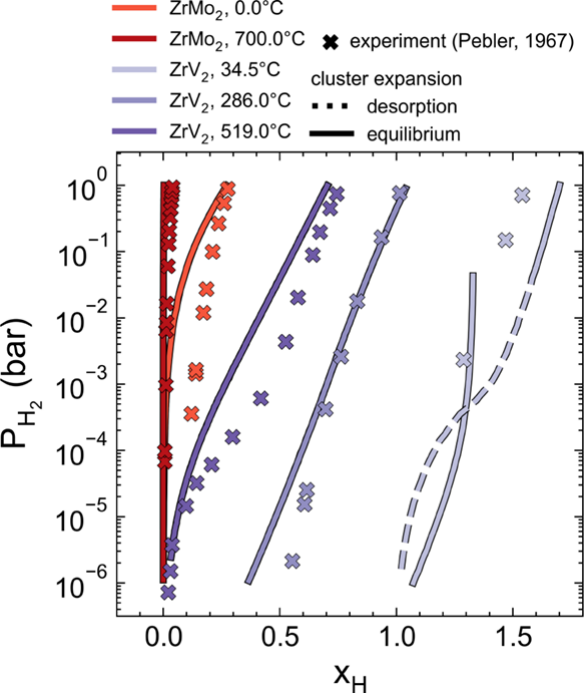
Pressure–composition isotherms (PCT) for hydrogen dissolution into ZrMo_2_ and ZrV_2_ at several temperatures. Experimental data are taken from Pebler and Gulbransen.^22^

The calculations of Figure 7 show that significant amounts of hydrogen can be stored in ZrV_2_ at modest temperatures and pressures. At 34.5 °C, both simulation and experiment indicate high hydrogen uptake, with the predicted composition varying from *x*_H_ ≈ 1 at a partial pressure of 10^–6^ bar to *x*_H_ > 1.5 at 1 bar. At elevated temperatures, the hydrogen concentration in ZrV_2_ is significantly reduced. For instance, at a partial pressure of 10^–6^ bar, the predicted composition decreases to *x*_H_ ≈ 0.5 at 286 °C and to nearly zero at 519 °C. These results suggest that extracting hydrogen from the ZrV_2_ Laves phase requires either extremely low partial pressures at room temperature or heating the compound to promote desorption.

Very little hydrogen is absorbed by ZrMo_2_ at low temperatures across all simulated partial pressures in Figure 7. At 0 °C, our model suggests that high partial pressures of 10–100 bar are required to achieve a hydrogen composition of *x*_H_ ≈ 0.5. At 700 °C very dilute concentrations of hydrogen are predicted to be accommodated within the C15 ZrMo_2_ phase.

Figure 7 displays slight discrepancies between theory and experiment for the pressure–composition isotherm of ZrV_2_ at 34.5 °C. The differences could arise from several sources. Two computed isotherms are shown in Figure 7 for ZrV_2_ at a temperature of 34.5 °C. The equilibrium curve (solid line) will be attained if equilibrium is maintained throughout the experiment. Figure 7 also depicts a metastable curve (dashed line) that is obtained by computationally desorbing hydrogen from a hydrogen-rich structure. This pressure–composition isotherm can be viewed as the degree of metastability that can be tolerated by the ZrV_2_ hydride with a large hydrogen composition before it undergoes a phase transition. The metastable curve is especially relevant when hydrogen dissolution may be kinetically limited in experiments. The results of Figure 7 suggest that the larger hydrogen compositions and partial pressures achieved in the experiments of Pebler and Gulbransen^22^ may be due to kinetic factors. In fact, kinetically hindered hydrogen desorption has been reported in another study^12^ even at a temperature of 100 °C. Quantitative discrepancies between theory and experiment may also arise due to limitations in the free-energy model such as missing vibrational and anharmonic entropy contributions or errors in DFT. Nevertheless, Figure 7 demonstrates excellent general agreement between the theoretical and experimental pressure–composition isotherms.

The relationship between hydrogen pressures and compositions shown in Figure 7 can be correlated with the DFT formation energies of Figure 3. The significantly more negative formation energies for hydrogen in ZrV_2_ compared to ZrMo_2_ indicate a stronger H-metal bond in the vanadium containing Laves phase. This stronger binding in ZrV_2_ explains the high hydrogen concentrations observed at 34.5 °C, even at a low pressure of 10^–6^ bar. Conversely, the weak interactions in ZrMo_2_ result in negligible hydrogen dissolution even at a pressure of 1 bar.

The distribution of hydrogen over the A_2_B_2_ and AB_3_ tetrahedral sites in ZrV_2_ at finite temperature as computed with the atomistic model and Monte Carlo simulations is shown in Figure 8. The figure displays the equilibrium concentrations and the concentrations along metastable pathways corresponding to hydrogen absorption and desorption. For hydrogen compositions below *x*_H_ ≈ 0.6, hydrogen occupies only the A_2_B_2_ tetrahedra. Above this threshold, the AB_3_ sites begin to populate. This behavior can be understood by considering the zero-Kelvin formation energies of Figure 3. While configurations with mixed-site occupancy are not ground states at 0 K, several such orderings lie only slightly above the convex hull. At finite temperatures, these states become thermally accessible as the gain in configurational entropy offsets the small energy penalty of occupying both tetrahedral sites. At higher hydrogen compositions, a larger fraction of the AB_3_ site is predicted to be occupied. Experimental measurements of site occupancies from several studies^23,40,45,46^ are also shown in Figure 8. As the experiments are performed at several temperatures ranging from 22–67 °C, the results from our computer simulations cannot be directly compared with experiments. However, the trends predicted by theory are in agreement with the experimental values of hydrogen site occupancy in the ZrV_2_ Laves phase.

**Figure 8:**
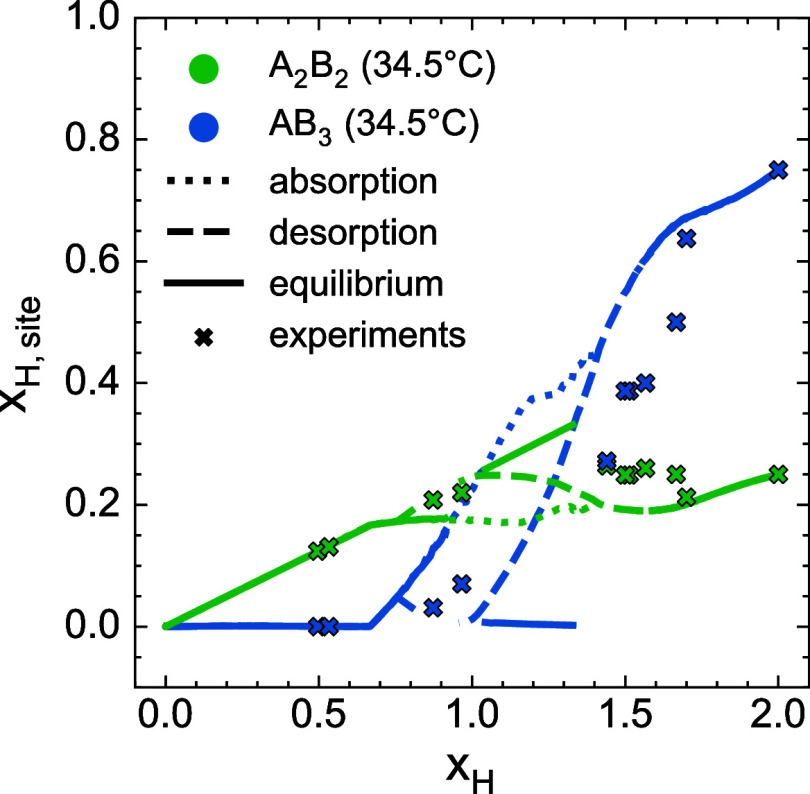
Comparison between computational predictions (lines) and experimental measurements (markers) of hydrogen concentrations in AB_3_ and A_2_B_2_ tetrahedra in ZrV_2_. Site occupancies are computed at 34.5 °C. Experimental site occupancies are from experiments^23,40,45,46^ performed across a temperature range of 22–67 °C.

## Discussion

Our first-principles calculations reveal fundamentally different hydrogen absorption behavior in the prototypical ZrMo_2_ and ZrV_2_ C15 Laves phases. ZrMo_2_ is predicted to accommodate hydrogen exclusively within A_2_B_2_ tetrahedral interstices. The weak bonding between the metal and hydrogen results in large pressures being needed to achieve hydrogen uptake (Figure 3). In contrast, the stronger binding between hydrogen and ZrV_2_ facilitates high hydrogen concentrations even at low hydrogen pressures and temperatures. The larger hydrogen capacity of ZrV_2_ is due to the simultaneous occupation of both A_2_B_2_ and AB_3_ interstitial sites at elevated temperatures. Our finite-temperature simulations reveal that both the A_2_B_2_ and AB_3_ sites remain partially occupied at 34.5 °C (Figure 8) similar to previous experiments. Overall, the predicted site occupancies and pressure–composition–temperature behavior from our computational model agree well with experimental results (Figures 7 and 8).

Previous experiments on the hydrides of ZrV_2_ have ascribed the emergence of an unexpected low-energy neutron peak to the presence of pairs of hydrogen atoms at distances approaching 1.6 Å.^12^ We performed finite-temperature simulations at a fixed hydrogen composition of $x_{H}=\frac{4}{3}$ to investigate the variation in site and pair occupancies with temperature (Figure 9). As shown in Figure 9a, ZrV_2_H_4_ forms a low-temperature ordered phase where hydrogen atoms exclusively occupy the A_2_B_2_ tetrahedral sites. This arrangement is identical to the ground state ordering found with DFT (Figures 3 and 4). As temperature increases, hydrogen atoms disorder and simultaneously occupy both A_2_B_2_ and AB_3_ sites. The order–disorder temperature shown in Figure 9a represents an upper bound on the true phase transition temperature due to hysteresis effects in canonical Monte Carlo simulations.

**Figure 9:**
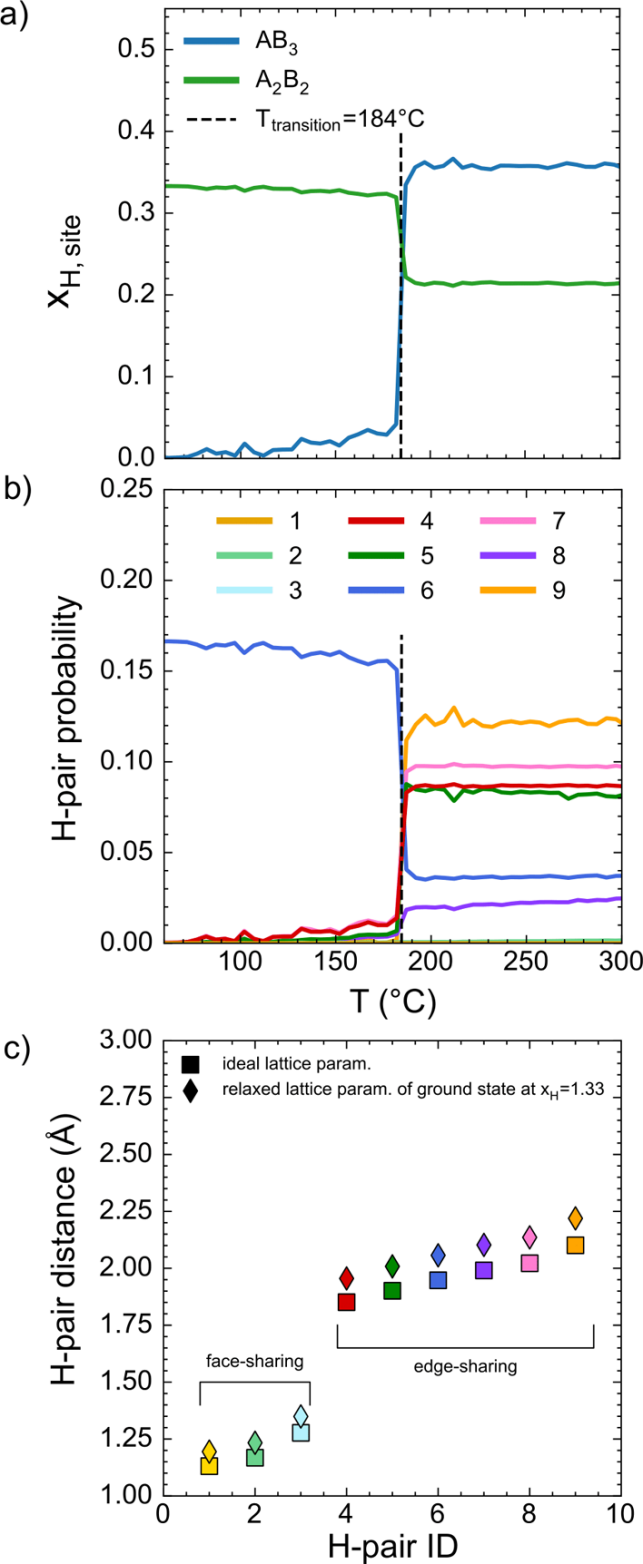
Monte Carlo simulations at a fixed composition of ZrV_2_H_4_ showing (a) site occupancies of hydrogen in AB_3_ and A_2_B_2_ tetrahedral sites, (b) pair cluster concentrations as a function of temperature, and (c) pair cluster distances in a pure ZrV_2_ Laves phase (square markers) and in a Laves phase with the lattice parameters of the ZrV_2_H_4_ ground state (diamond markers).

The experiments by Didisheim et al.^42^ have reported an order–disorder phase transition in deuterated ZrV_2_. The simulations of Figure 9a are in excellent agreement with these experiments. For instance, both our simulations and previous experiments^42^ reveal a tetragonal low-temperature phase containing hydrogen atoms distributed over A_2_B_2_ interstices. Analysis of hysteresis effects within our Monte Carlo simulations suggests an order–disorder phase transition close to room temperature - in agreement with the experimental measurements of ref 42. The predicted distribution of hydrogen atoms over both A_2_B_2_ and AB_3_ interstices at elevated temperatures in Figure 9a are also in agreement with experiment. The close alignment between the theoretical results of Figure 9 and previous experiments^42^ suggest that the computational model accurately captures the chemical interactions of hydrogen within ZrV_2_.

Figure 9b shows the temperature-dependent occupancy of the first nine nearest-neighbor hydrogen pair clusters in ZrV_2_H_4_. The first three clusters span hydrogen sites between face-sharing tetrahedra, while the remaining clusters, labeled 4–9, correspond to pairs of sites between edge-sharing tetrahedra. Figure 9c compares the pair distances in the hydrogen-free intermetallic with those in the ground state ZrV_2_H_4_ structure. All pair distances are elongated when the hydrogen composition increases. This lattice expansion is consistent with the linear variation in volume with hydrogen concentration shown in Figure S2. The distances presented in Figure 9c are for an ideal C15 structure, and do not account for any local atomic relaxations.

The results of our rigorous first-principles statistical mechanics simulations shown in Figure 9b suggest that hydrogen sites located at distances of ≈2.0 Å are populated at elevated temperatures. At low temperatures, the shortest H–H distance is ≈2.1 Å. Above the order–disorder transition, entropy drives the occupation of several additional pair clusters. The shortest of these newly occupied pairs, labeled 4 in Figure 9, has a H–H distance of ≈2.0 Å. The first three nearest pair clusters, with hydrogen pairs located at distances less than 1.6 Å, are not found to have a significant concentration in the simulations of Figure 9b.

In contrast to the study of Borgschulte et al.,^12^ our finite-temperature simulations of Figure 9 do not predict an appreciable population of H–H pairs with distances of 1.6 Å in ZrV_2_. Though our simulations reveal some finite-temperature H–H pairs that are located at a distance close to 2.0 Å, this does not constitute a strong violation of the Switendick criterion for stable hydrides. The simulations of Figure 9 do not account for thermal effects such as lattice expansion, and local relaxations that might occur in the disordered phase. Additionally, as shown in Figure S2, the volumes of the ZrV_2_ hydrides predicted by DFT are slightly smaller than experimentally measured volumes. This likely arises due to well-known limitations of the PBE functional used in our calculations. Given these uncertainties, the population of the fourth nearest H–H pair in our simulations does not present a clear violation of the Switendick criterion.

To investigate the absence of H–H pairs at distances lower than 2.1 Å, we computed point defect energies in the 0 K ground state with a composition of $x_{H}=\frac{4}{3}$ . The calculations used a supercell containing 8 ZrV_2_ formula units to minimize defect interactions. We created point defects by moving a single hydrogen atom from its ground state position to a vacant site less than 2.1 Å from another occupied site. After fully relaxing the atomic positions with DFT, the defect formation energies are predicted to lie between 0.6 to 1.0 eV, as shown in Figure 10. Interestingly, several distinct initial configurations relaxed into similar high-energy states where the closest H–H distance was ≈1.5 Å. The defect energies of Figure 10 are comparable to the value of ≈0.5 eV reported by Borgschulte et al.^12^ for a similar defect. The high point defect energies indicate that the concentration of such defects is negligible at room temperature and remains low (≈10^–4^) even at 400 °C. The large defect formation energies for H–H pairs occupying face-sharing tetrahedra are in agreement with the lack of a significant population of face-sharing H–H pairs in the finite-temperature results of Figure 9. The low-energy neutron scattering peak observed by Borgschulte et al. could originate from kinetically constrained, nonequilibrium states or may have a different origin. Further investigation is required to clarify the source of this feature.

**Figure 10:**
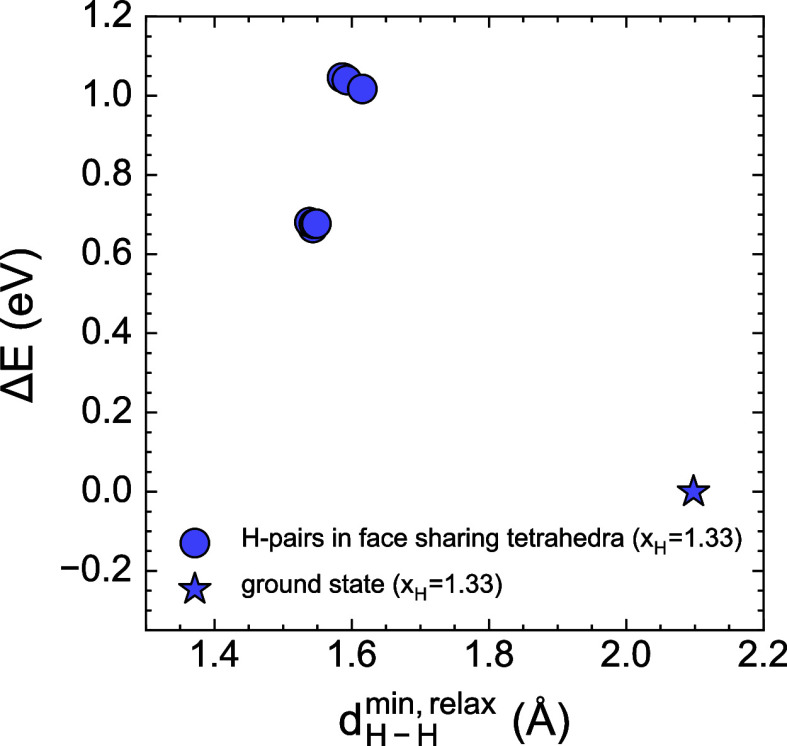
Defect energies relative to the ZrV_2_H_4_ ground state as a function of the H–H pair distances computed within a supercell containing 8 formula units of ZrV_2_.

The results of Figures 7 and 9 suggest that enabling hydrogen dissolution into multiple interstitial sites could significantly enhance the hydrogen storage capacity of Laves phases. In ZrV_2_, the finite-temperature simulations of Figures 8 and 9 show that hydrogen occupies both AB_3_ and A_2_B_2_ interstices. The simultaneous occupation of both tetrahedral interstices enables a high capacity of almost *x*_H_ = 1.5 at ambient conditions. In contrast, the storage capacity of ZrMo_2_ is lower as hydrogen dissolves only within the A_2_B_2_ sites.

The trends in site preference for hydrogen dissolution shown in Figure 3 can be predicted from the dilute dissolution energies of hydrogen. Table 1 lists the dilute dissolution energies across the three interstitial sites in ZrV_2_ and ZrMo_2_ calculated relative to the intermetallic compound and a hydrogen molecule at zero-Kelvin. B_4_ tetrahedra are found to be energetically unfavorable in both compounds. In ZrMo_2_, the A_2_B_2_ site is strongly preferred over the AB_3_ site by nearly 200 meV. In contrast, the dissolution energies for the A_2_B_2_ and AB_3_ site in ZrV_2_ differ by only 0.09 eV. The distinct site preferences observed at dilute hydrogen concentrations are consistent with the formation energies across the full range of hydrogen compositions shown in Figure 3. The similar hydrogen dissolution energies for the two interstitial sites in ZrV_2_ are likely governed by chemical interactions rather than the size of the interstices. Varying the lattice parameter does not cause the dissolution energy differences in ZrMo_2_ to resemble those in ZrV_2_ (Figure S3), indicating that the size of the interstitial site is not the dominant factor. Instead, these energy differences are more sensitive to the chemical composition of the Laves phase. For several AB_2_ Laves phases with Zr at the A-site, the choice of the B-site transition metal governs the energy differences between the interstitial sites (Figure S4). Period 4 transition metals from groups 6–9 yield similar dissolution energies for both A_2_B_2_ and AB_3_ interstices, whereas most other transition elements cause a strong energetic preference for the A_2_B_2_ site. This trend likely reflects differences in the local bonding between hydrogen and the surrounding metal atoms.

**Table 1:** Hydrogen Dissolution Energies (eV) for a Single Hydrogen within a Supercell Containing 54 Formula Units of AB_2_

laves compound	*E*_d,A_2_B_2__ (eV)	*E*_d,AB_3__ (eV)	*E*_d,B_4__ (eV)
ZrMo_2_	–0.228	0.008	0.706
ZrV_2_	–0.744	–0.657	–0.320

The results of Table 1 and Figure 3 suggest that Laves phases with similar hydrogen dissolution energies for the A_2_B_2_ and AB_3_ interstices will accommodate higher concentrations of hydrogen. Motivated by this, we performed high-throughput DFT calculations to search for binary C15 Laves phases capable of incorporating hydrogen within multiple interstitial sites. First, we relaxed binary C15 compounds comprised of pairs of elements listed in Figure 11 to their 0 K ground state. We selected all compounds with negative formation energies for further analysis, as well as several refractory compounds with slightly positive formation energies. Refractory Laves phases are included in our high-throughput search as they have recently been shown to be promising candidates for hydrogen storage applications.^15^ For the 421 candidate compounds, we computed the hydrogen dissolution energy for each of the three interstitial site types using a supercell containing 54 formula units of the Laves compound. To ensure structural stability, we discarded any calculations where the average atomic displacement upon relaxation exceeded 0.2 Å. This filtering resulted in a final data set of 963 distinct dissolution energies.

**Figure 11:**
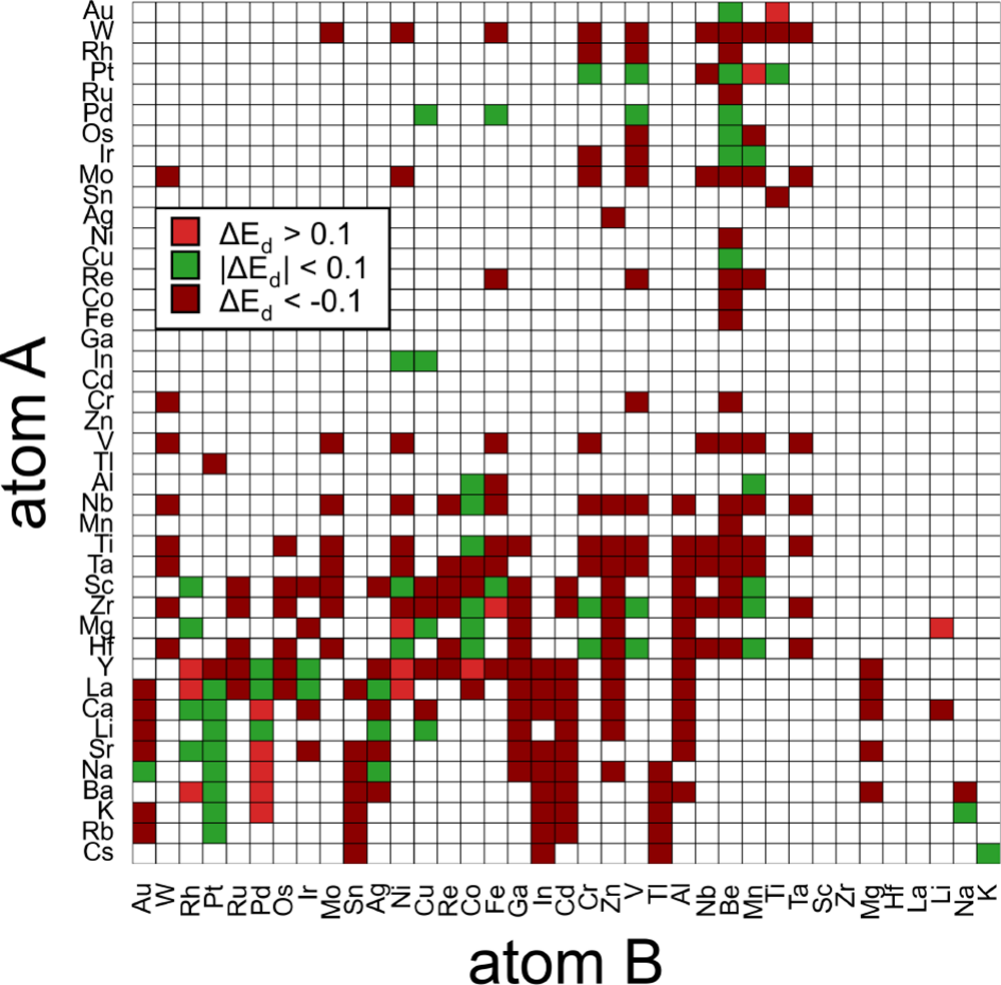
Heat map of dissolution energy differences between A_2_B_2_ and AB_3_ sites as computed with DFT in binary Laves phases with a stoichiometry of AB_2_. Chemistries marked with a dark red prefer to dissolve hydrogen within the A_2_B_2_ site and light red prefer AB_3_ environments. The dissolution energies for both sites are within 0.1 eV for the chemistries marked in green. Compounds labeled with white either had Laves phases with positive formation energies, underwent large structural relaxations, or did not result in a converged DFT calculation. Dissolution energies are computed within a supercell containing 54 formula units of AB_2_.

Figure 11 illustrates the energy difference for hydrogen occupying a site within either a A_2_B_2_ or a AB_3_ coordinated tetrahedron in binary intermetallic C15 Laves phases. Intermetallic compounds where the A_2_B_2_ tetrahedral site is significantly more favorable than the AB_3_ site are labeled in dark red. Compounds where the AB_3_ site is more stable are labeled in light red. Intermetallic compounds where the energy differences for hydrogen occupation are less than 0.1 eV are shown in green.

The results of Figure 11 show that the vast majority of intermetallic compounds preferentially accommodate hydrogen within A_2_B_2_ coordinated tetrahedra. This aligns with previous experimental and theoretical work, where the A_2_B_2_ tetrahedra are typically assumed to be the only interstitial network to be populated by hydrogen in the C15 Laves phase. Our calculations also reveal a small number of compounds, particularly those containing late transition metals, that prefer to host hydrogen in the AB_3_ site. Interestingly, the high-throughput search predicted 15 Laves phase compounds to have an energetic preference for hydrogen dissolution within the B_4_ tetrahedron.

Several intermetallic compounds, labeled in green in Figure 11, show a similar energetic preference for hydrogen dissolution into the AB_3_ and A_2_B_2_ sites. A detailed list of these compounds is provided in Table 2. An ideal candidate for hydrogen storage must meet at least two important thermodynamic criteria. First, the hydrogen dissolution energy should be slightly negative to allow for hydrogen absorption and desorption under ambient conditions. Additionally, based on our findings for ZrV_2_, the hydrogen dissolution energy difference between AB_3_ and A_2_B_2_ should be close to zero. This allows for the occupation of multiple sites and could enable higher hydrogen storage capacities. Several other considerations must be satisfied for practical applications, such as the reversibility of the hydrogen absorption and desorptions reactions, the plateau pressures for hydrogen absorption, the kinetics of hydrogen dissolution etc. We do not consider these additional requirements in this study.

**Table 2:** C15 Laves Phase Compounds with Dissolution Energy Differences between AB_3_ and A_2_B_2_ Tetrahedral Sites That Are within 0.1 eV^a^

AB_2_	*E*_d,A_2_B_2__ (eV)	*E*_d,AB_3__ (eV)	Δ*E*_d_ (eV)	*E*_f_^AB_2_^ (eV/f.u.)
LiAg_2_	0.348	0.447	–0.100	–0.285
HfNi_2_	0.133	0.231	–0.098	–1.446
HfV_2_	–1.693	–1.598	–0.095	0.099
PdV_2_	0.184	0.275	–0.092	–0.205
ZrV_2_	–0.744	–0.657	–0.087	0.131
OsBe_2_	1.325	1.411	–0.086	–0.811
HfCr_2_	–0.206	–0.124	–0.082	–0.344
NbCo_2_	0.192	0.272	–0.079	–0.482
IrMn_2_	0.765	0.844	–0.079	–0.176
ZrCr_2_	–0.252	–0.188	–0.064	–0.147
NaAu_2_	0.675	0.739	–0.063	–1.120
TiCo_2_	0.251	0.312	–0.061	–0.923
PtV_2_	0.551	0.612	–0.060	–0.933
IrBe_2_	1.284	1.341	–0.057	–1.267
ScMn_2_	–0.012	0.044	–0.056	–0.467
PdFe_2_	0.304	0.358	–0.054	–0.012
CuBe_2_	0.988	1.041	–0.052	–0.412
LiPt_2_	0.055	0.103	–0.047	–1.311
PtTi_2_	0.053	0.089	–0.036	–0.968
HfMn_2_	0.084	0.118	–0.034	–0.876
YIr_2_	0.363	0.391	–0.027	–2.450
CaPt_2_	0.378	0.403	–0.025	–2.804
MgRh_2_	0.005	0.029	–0.024	–0.717
PtBe_2_	0.129	0.150	–0.021	–1.388
LaPt_2_	0.428	0.443	–0.016	–3.234
YPd_2_	0.200	0.214	–0.014	–2.415
KNa_2_	0.060	0.073	–0.013	–0.019
ZrMn_2_	0.017	0.030	–0.013	–0.689
NaPt_2_	0.033	0.044	–0.011	–1.105
PtCr_2_	0.833	0.840	–0.007	–0.166
NaAg_2_	0.507	0.509	–0.002	–0.307
InNi_2_	–0.042	–0.041	–0.001	–0.084
LiCu_2_	0.229	0.228	0.000	–0.214
AlCo_2_	0.408	0.407	0.001	–0.412
ScRh_2_	0.255	0.250	0.005	–2.075
InCu_2_	0.604	0.594	0.010	–0.034
ScFe_2_	–0.026	–0.036	0.010	–0.825
SrPt_2_	0.265	0.251	0.014	–2.544
BaPt_2_	0.218	0.196	0.021	–2.136
CsK_2_	0.024	–0.007	0.031	–0.012
MgCu_2_	0.676	0.643	0.033	–0.443
AlMn_2_	0.668	0.635	0.033	–0.283
LaAg_2_	–1.402	–1.438	0.036	–0.534
ScNi_2_	0.150	0.111	0.038	–1.562
CaRh_2_	–0.239	–0.278	0.038	–1.198
AuBe_2_	0.907	0.868	0.039	–0.375
PdBe_2_	0.893	0.847	0.046	–1.196
PdCu_2_	0.445	0.393	0.052	–0.187
KPt_2_	–0.130	–0.186	0.056	–0.551
LaIr_2_	0.268	0.210	0.058	–2.183
HfCo_2_	0.191	0.122	0.069	–1.109
LiPd_2_	–0.038	–0.110	0.072	–0.708
LaPd_2_	0.236	0.159	0.078	–2.369
ZrCo_2_	0.199	0.116	0.083	–0.978
SrRh_2_	–0.399	–0.483	0.084	–0.685
MgCo_2_	0.277	0.188	0.089	–0.031
RbPt_2_	–0.229	–0.326	0.097	–0.071

a Dissolution energies in the individual tetrahedral sites are computed within a supercell containing 54 formula units of AB_2_ and are listed along with the energy differences and the formation energy of the C15 Laves phase.

The high-throughput search of Figure 11 and Table 1 identified several viable C15 Laves phase candidates for hydrogen storage, including CaRh_2_, HfCr_2_, KPt_2_, RbPt_2_, and ZrCr_2_. Of these, HfCr_2_ and ZrCr_2_ are particularly attractive as they are composed of nonprecious metals. We confirm the viability of ZrCr_2_ for hydrogen storage by computing the 0 K formation energies of hydrogen orderings in the C15 intermetallic compound. The results of Figure 12 confirm that the A_2_B_2_ and AB_3_ sites are energetically competitive in ZrCr_2_ across the full range of hydrogen compositions. In fact, the ground state at *x*_H_ = 1.5 is predicted to contain hydrogen in both tetrahedral site types. The formation energies of the orderings containing hydrogen in ZrCr_2_ are comparable to those of ZrMo_2_.

**Figure 12:**
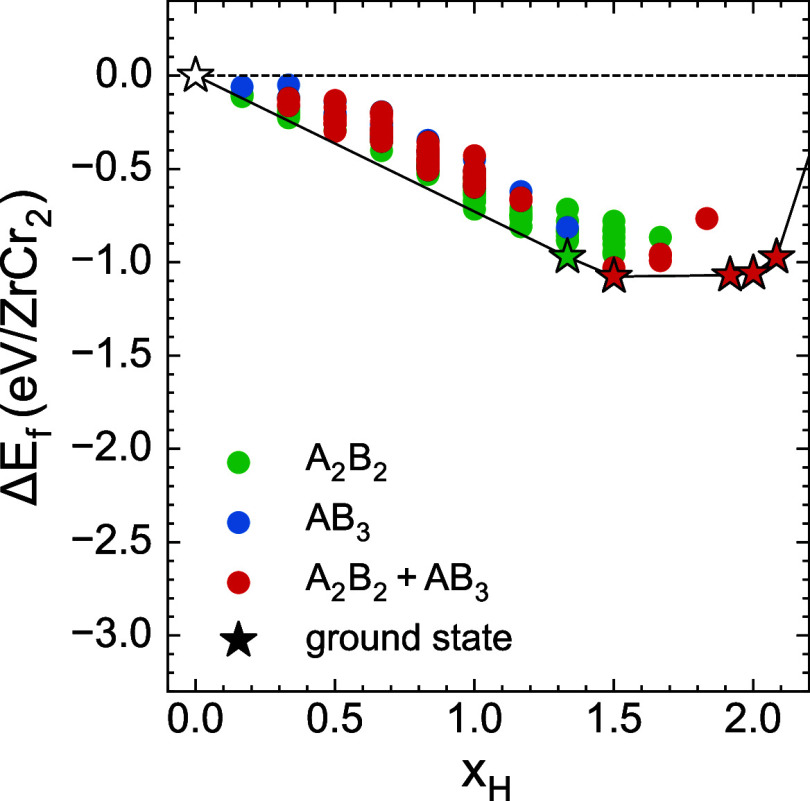
DFT formation energies of hydrogen-vacancy orderings in ZrCr_2_ colored by tetrahedral environments occupied by hydrogen.

The high-throughput screening results (Table 2 and Figure 11) also provide guidance for designing multicomponent alloys for hydrogen storage. Recent efforts have focused on synthesizing single-phase multicomponent Laves phases with high hydrogen storage capacities.^15^ Table 2 and Figure 11 can serve as a starting point to tune material chemistries and improve storage capacity by optimizing hydrogen absorption energies, dissolution energies in tetrahedral sites, and intermetallic phase stability.

## Conclusions

In this study we have presented a rigorous first-principles analysis of the thermodynamics of hydrogen absorption into binary C15 Laves phases. The prototypical ZrMo_2_ compound accommodates hydrogen within the A_2_B_2_ coordinated tetrahedra, while the ZrV_2_ compound distributes H atoms over both A_2_B_2_ and AB_3_ sites. The availability of more interstitial sites for hydrogen uptake within the ZrV_2_ compound enables a higher storage capacity. Pressure–composition isotherms computed from our ab initio model are in excellent agreement with previous experiments for both intermetallic compounds. The ZrV_2_H_4_ compound is predicted to undergo an order–disorder phase transition at elevated temperatures. Pairs of hydrogen atoms within this hydride are separated by a distance very close to the Switendick criterion for stable hydrides. High-throughput calculations of dilute hydrogen dissolution energies across stable intermetallic binary C15 Laves phases revealed several promising hydrogen storage candidates that can accommodate H within multiple interstitial sites. The results of this study provide a candidate set of binary C15 Laves phases that could be interesting for further experiments to enable room temperature stationary hydrogen storage at moderate hydrogen partial pressures.

## Supplementary Material



## Data Availability

The data associated with this study is available on the Materials Cloud Platform at https://doi.org/10.24435/materialscloud:xf-s7.

## Notes

Derivation of the free-energy model for hydrogen absorption within the Laves phase; zero-point energies of hydrogen; and variation in the volume of ZrV_2_ with hydrogen composition and chemical trends in the hydrogen dissolution energies (PDF)

## References

[1] Sartori S., O’Hayre R., Shao Z. (2024). Materials for green hydrogen production,
storage, and conversion. MRS Bull..

[2] Allendorf M. D., Stavila V., Snider J. L., Witman M., Bowden M. E., Brooks K., Tran B. L., Autrey T. (2022). Challenges to Developing
Materials for the Transport and Storage of Hydrogen. Nat. Chem..

[3] Cho Y., Cho H., Cho E. S. (2023). Nanointerface Engineering of Metal
Hydrides for Advanced
Hydrogen Storage. Chem. Mater..

[4] Bellosta
Von Colbe J. (2019). Application of Hydrides in Hydrogen Storage
and Compression: Achievements, Outlook and Perspectives. Int. J. Hydrogen Energy.

[5] Hirscher M. (2020). Materials for Hydrogen-Based
Energy Storage – Past, Recent
Progress and Future Outlook. J. Alloys Compd..

[6] Stein F., Palm M., Sauthoff G. (2004). Structure
and stability of Laves
phases. Part I. Critical assessment of factors controlling Laves phase
stability. Intermetallics.

[7] Stein F., Leineweber A. (2021). Laves Phases:
A Review of Their Functional and Structural
Applications and an Improved Fundamental Understanding of Stability
and Properties. J. Mater. Sci..

[8] Kolli S. K., Natarajan A. R., Thomas J. C., Pollock T. M., Van der
Ven A. (2020). Discovering Hierarchies among Intermetallic Crystal Structures. Physical Review Materials.

[9] Natarajan A. R., Van der Ven A. (2018). Connecting
the Simpler Structures to Topologically
Close-Packed Phases. Phys. Rev. Lett..

[10] Ferro, R. ; Saccone, A. Intermetallic chemistry; Elsevier, 2008; 13.

[11] Yartys V. A., Lototskyy M. V. (2022). Laves type intermetallic compounds as hydrogen storage
materials: A review. J. Alloys Compd..

[12] Borgschulte A., Terreni J., Billeter E., Daemen L., Cheng Y., Pandey A., Łodziana Z., Hemley R. J., Ramirez-Cuesta A. J. (2020). Inelastic
Neutron Scattering Evidence for Anomalous H–H Distances in
Metal Hydrides. Proc. Natl. Acad. Sci. U. S.
A..

[13] Switendick A. C. (1979). Band Structure
Calculations for Metal Hydrogen Systems∗. Zeitschrift f ü r Physikalische Chemie.

[14] Zepon G., Silva B. H., Zlotea C., Botta W. J., Champion Y. (2021). Thermodynamic
Modelling of Hydrogen-Multicomponent Alloy Systems: Calculating Pressure-Composition-Temperature
Diagrams. Acta Mater..

[15] Mohammadi A., Ikeda Y., Edalati P., Mito M., Grabowski B., Li H.-W., Edalati K. (2022). High-Entropy
Hydrides for Fast and
Reversible Hydrogen Storage at Room Temperature: Binding-Energy Engineering
via First-Principles Calculations and Experiments. Acta Mater..

[16] Witman M. D. (2023). Towards Pareto Optimal High Entropy Hydrides *via* Data-Driven Materials Discovery. Journal of
Materials Chemistry A.

[17] Agafonov A., Pineda-Romero N., Witman M., Nassif V., Vaughan G. B., Lei L., Ling S., Grant D. M., Dornheim M., Allendorf M., Stavila V., Zlotea C. (2024). Destabilizing High-Capacity High
Entropy Hydrides via Earth Abundant Substitutions: From Predictions
to Experimental Validation. Acta Mater..

[18] Agafonov A., Pineda-Romero N., Witman M. D., Enblom V., Sahlberg M., Nassif V., Lei L., Grant D. M., Dornheim M., Ling S., Stavila V., Zlotea C. (2025). Promising Alloys for
Hydrogen Storage in the Compositional Space of (TiVNb)_100–*x*
_(Cr,Mo)_
*x*
_ High-Entropy
Alloys. ACS Appl. Mater. Interfaces.

[19] Shoemaker D. P., Shoemaker C. B. (1979). Concerning Atomic Sites and Capacities
for Hydrogen
Absorption in the AB2 Friauf-Laves Phases. Journal
of the Less Common Metals.

[20] Shaltiel D. (1980). Hydride Properties
of AB2 Laves-Phase Compounds. Journal of the
Less Common Metals.

[21] Ivey D., Northwood D. (1986). Hydrogen site occupancy in AB2 Laves
phases. Journal of the Less Common Metals.

[22] Pebler, A. ; Gulbransen, E. Equilibrium studies on the systems ZrCr 2-H 2, ZrV 2-H 2, and ZrMo 2-H 2 between 0 and 900 C. AIME Met. Soc. Trans. 1967, 239

[23] Didisheim J.-J., Yvon K., Fischer P., Shaltiel D. (1980). The Deuterium Site
Occupation in ZrV2Dx as a Function of the Deuterium Concentration. Journal of the Less Common Metals.

[24] Fruchart D., Rouault A., Shoemaker C., Shoemaker D. (1980). Neutron Diffraction
Studies of the Cubic ZrCr2Dx and ZrV2Dx (Hx) PHASES. Journal of the Less Common Metals.

[25] Kresse G., Hafner J. (1993). *Ab Initio* Molecular Dynamics for Liquid
Metals. Phys. Rev. B.

[26] Kresse G., Furthmüller J. (1996). Efficient
Iterative Schemes for *Ab Initio* Total-Energy Calculations
Using a Plane-Wave Basis Set. Phys. Rev. B.

[27] Blöchl P. E. (1994). Projector
Augmented-Wave Method. Phys. Rev. B.

[28] Perdew J. P., Burke K., Ernzerhof M. (1996). Generalized
Gradient Approximation
Made Simple. Phys. Rev. Lett..

[29] Sanchez J., Ducastelle F., Gratias D. (1984). Generalized Cluster Description of
Multicomponent Systems. Physica A: Statistical
Mechanics and its Applications.

[30] Fontaine, D. D. Solid State Physics; Elsevier, 1994; 47; 33–176.

[31] Müller Y. L., Natarajan A. R. (2025). Constructing Multicomponent Cluster
Expansions with
Machine-Learning and Chemical Embedding. npj
Comput. Mater..

[32] Gunda N. S. H., Van der Ven A. (2018). First-Principles
Insights on Phase Stability of Titanium
Interstitial Alloys. Physical Review Materials.

[33] Gunda N. S. H., Puchala B., Van der
Ven A. (2018). Resolving Phase Stability in the
Ti-O Binary with First-Principles Statistical Mechanics Methods. Physical Review Materials.

[34] Gunda N. S. H., Van der Ven A. (2020). Understanding
the Interactions between Interstitial
and Substitutional Solutes in Refractory Alloys: The Case of Ti-Al-O. Acta Mater..

[35] Li J., Ober D., Van Der Ven A. (2024). Phase Stability in the Hf-N and Zr-N
Systems. Phys. Rev. Mater..

[36] Puchala B., Van Der Ven A. (2013). Thermodynamics
of the Zr-O System from First-Principles
Calculations. Phys. Rev. B.

[37] Xu Q., Van Der Ven A. (2007). First-Principles
Investigation of Metal-Hydride Phase
Stability: The Ti-H System. Phys. Rev. B.

[38] Puchala B., Thomas J. C., Natarajan A. R., Goiri J. G., Behara S. S., Kaufman J. L., Van der
Ven A. (2023). CASM — A Software Package
for First-Principles Based Study of Multicomponent Crystalline Solids. Comput. Mater. Sci..

[39] Van
der Ven A., Thomas J., Puchala B., Natarajan A. (2018). First-Principles
Statistical Mechanics of Multicomponent Crystals. Annu. Rev. Mater. Res..

[40] Didisheim J.-J., Yvon K., Shaltiel D., Fischer P., Bujard P., Walker E. (1979). The Distribution of
the Deuterium Atoms in the Deuterated
Cubic Laves-Phase ZrV2D4·5. Solid State
Commun..

[41] Fruchart D., Rouault A., Shoemaker C. B., Shoemaker D. P. (1980). Neutron
Diffraction Studies of ZrCr2Dx and ZrV2Dx (Hx). Physica Status Solidi (a).

[42] Didisheim J.-J., Yvon K., Fischer P., Tissot P. (1981). Order-Disorder Phase
Transition in ZrV2D3.6. Solid State Commun..

[43] Irodova A., Glazkov V., Somenkov V., Shilstein S. (1981). Hydrogen Ordering
in the Cubic Laves Phase HfV2. Journal of the
Less Common Metals.

[44] Thomas J. C., Natarajan A. R., Van der Ven A. (2021). Comparing
Crystal Structures with
Symmetry and Geometry. npj Comput. Mater..

[45] Somenkov V., Irodova A. (1984). Lattice structure and phase transitions
of hydrogen
in intermetallic compounds. Journal of the Less
Common Metals.

[46] Bogdanova A. (2006). Neutron diffraction
study of phase transitions in highly concentrated hydrogen solid solutions
ZrV 2 D x (4< x< 5). Phys. Solid State.

