# Supplementary material for: First-Principles Thermodynamics of Hydrogen Absorption in Binary C15 Laves Phases
Source: Chem Mater. 2026 Jan 7;38(2):683–93. doi: 10.1021/acs.chemmater.5c01925 (PMC12854679; doi:10.1021/acs.chemmater.5c01925)
Supplement: Supplementary file 1 [file cm5c01925_si_001.pdf]

# Supporting Information:
# First-principles thermodynamics of hydrogen absorption in binary C15 Laves phases

Claire A. Paetsch[1,2] and Anirudh Raju Natarajan[1,2,*]

[1]*Laboratory of materials design and simulation (MADES), Institute of Materials, École Polytechnique Fédérale de Lausanne, CH-1015 Lausanne, Switzerland*

[2]*National Centre for Computational Design and Discovery of Novel Materials (MARVEL), École Polytechnique Fédérale de Lausanne, CH-1015 Lausanne, Switzerland*

## S1. THERMODYNAMICS OF HYDROGEN ABSORPTION

The Gibbs free energy of an intermetallic compound ($g^f_\alpha(T, x_H)$) containing $N_M$ atoms of metal species, $N_H$ atoms of hydrogen at a temperature $T$ and hydrogen composition $x_H = \frac{N_H}{N_M}$ relative to the hydrogen-free compound and gaseous hydrogen is given by:

$$g^f_\alpha(T, x_H) = g_\alpha(T, x_H) - g_\alpha(T, x_H = 0) - \frac{x_H}{2} g_{H_2}(T, p^0_{H_2}) \tag{S1}$$

where $g_\alpha = \frac{G_\alpha}{N_M}$ is the total free energy of $\alpha$ per metal atom, $g_{H_2}(T, p^0_{H_2})$ is the free energy of one molecule of hydrogen gas at a temperature $T$ and reference partial pressure $p^0_{H_2}$.

The free energies of the hydride, metal, and hydrogen molecule have contributions arising from configurational and vibrational entropy:

$$\begin{aligned} g_\alpha(T, x_H) = {} & g^{\text{config}}_\alpha(T, x_H) + g^{\text{vibration, M}}_\alpha(T, x_H) \\ & + g^{\text{vibration, H}}_\alpha(T, x_H) + g^{\text{vibration, M-H}}_\alpha(T, x_H) \end{aligned} \tag{S2}$$

$$g_\alpha(T, x_H = 0) = g_\alpha(T = 0, x_H = 0) + g^{\text{vibration, M}}_\alpha(T, x_H = 0) \tag{S3}$$

$$g_{H_2}(T, p^0_{H_2}) = g_{H_2}(T = 0, p^0_{H_2}) + g^{\text{vibration}}_{H_2}(T, p^0_{H_2}) \tag{S4}$$

where $g^{\text{config}}_\alpha$ is the free energy with entropy contributions arising from the various possible arrangements of hydrogen atoms within the interstitial network of the metal host, and $g^{\text{vibration}}$ are vibrational free energy contributions arising from vibrations of the hydrogen molecule in the gas phase ($g^{\text{vibration}}_{H_2}(T, p^0_{H_2})$), the metal host atoms ($g^{\text{vibration, M}}_\alpha(T, x_H)$), the hydrogen atoms within the metal host ($g^{\text{vibration, H}}_\alpha(T, x_H)$) and additional vibrational entropy due to the coupling between the metal host and absorbed hydrogen atoms ($g^{\text{vibration, M-H}}_\alpha(T, x_H)$).

We make several approximations to make it computationally tractable to estimate eq. (S1). The zero-Kelvin energy of $H_2$ is approximated to be the energy of a hydrogen molecule in vacuum. We neglect the effects of disorder on the metal sublattice, and the vibrational free energy contributions arising from the coupling between the metal and hydrogen sublattices. Further, we assume that the vibrational free energy of the metal atoms in the hydride is equal to the vibrational free energy of the pure metal host ($g^{\text{vibration, M}}_\alpha(T, x_H) \approx g^{\text{vibration}}_\alpha(T, x_H = 0)$). The vibrational free energy of hydrogen in the hydride and the vibrational free energy of the hydrogen molecule is approximated as:

$$g^{\text{vibration, H}}_\alpha(T, x_H) \approx g^{\text{ZPE,H}}_\alpha(x_H) + \Delta g^{\text{vibration,H}}_\alpha(T, x_H) \tag{S5}$$

$$g^{\text{vibration}}_{H_2}(T, p^0_{H_2}) = g^{\text{ZPE}}_{H_2}(p^0_{H_2}) + \Delta g^{\text{vibration}}_{H_2}(T, p^0_{H_2}) \tag{S6}$$

* anirudh.natarajan@epfl.ch

where $\Delta g_{\alpha}^{\text{vibration,H}}(T, x_H)$, and $\Delta g_{H_2}^{\text{vibration}}(T, p^0_{H_2})$ are the vibrational free energy differences between 0 K and a finite temperature $T$ for the absorbed hydrogen and the hydrogen molecule and $g_{\alpha}^{\text{ZPE,H}}(x_H)$ and $g_{H_2}^{\text{ZPE}}(p^0_{H_2})$ are the zero-point energies of hydrogen within the intermetallic compound and the gas phase, respectively. $\Delta g_{H_2}^{\text{vibration}}(T, p^0_{H_2})$ is computed from the experimental values of the free energies of a hydrogen molecule tabulated in the NIST-JANAF thermochemical database [S1]. Using these relations in eq. (S1), we get:

$$g_{\alpha}^{f}(T, x_H) = g_{\alpha}^{f,\text{config}}(T, x_H) + x_H(\overline{g}_{\alpha}^{\text{ZPE,H}}(x_H) - \frac{1}{2} g_{H_2}^{\text{ZPE}}(p^0_{H_2}) - \frac{1}{2}\Delta g_{H_2}^{\text{vibration}}(T, p^0_{H_2})) \tag{S7}$$

where the configurational free energy of hydrogen absorbed in $\alpha$ is $g_{\alpha}^{f,\text{config}}(T, x_H) = g_{\alpha}^{\text{config}}(T, x_H) - g_{\alpha}(T = 0, x_H = 0) - \frac{x_H}{2} g_{H_2}(T = 0, p^0_{H_2})$, and $\overline{g}_{\alpha}^{\text{ZPE,H}}(x_H) = \frac{g_{\alpha}^{\text{ZPE,H}}(x_H)}{x_H}$ The vibrational free energy arising from hydrogen vibrations within the metal host is neglected eq. (S7) as this term is likely to be small in comparison with the vibrational free energy of the hydrogen gas. Further, we approximate the ZPE per hydrogen atom in the metal host, $\overline{g}_{\alpha}^{\text{ZPE,H}}(x_H)$ to be a constant value.

The chemical potential of hydrogen that is dissolved in a host metal is then given by:

$$\mu_{H,\alpha}(T, x_H) = \left(\frac{\partial g_{\alpha}}{\partial x_H}\right)_{T,N_M} \tag{S8}$$

When the hydride is in equilibrium with hydrogen gas at a partial pressure of $p_{H_2}$, thermodynamic equilibrium requires that:

$$\mu_{H,\alpha}(T, x_H) = \frac{1}{2}\mu_{H_2}(T, p_{H_2}) \tag{S9}$$

Substituting eqs. (S7) and (S8) in eq. (S9) we get:

$$p_{H_2} = p^0_{H_2} \exp\left(2\frac{\mu_{H,\alpha}^{\text{config}} + \Delta g_H^{vibrational}(T, p^0_{H_2})}{k_B T}\right) \tag{S10}$$

where $\mu_{H,\alpha}^{\text{config}} = \left(\frac{\partial g_{\alpha}^{f,\text{config}}}{\partial x_H}\right)_{T,N_M}$ and $\Delta g_H^{vibrational}(T, p^0_{H_2})$ is :

$$\Delta g_H^{vibrational}(T, p^0_{H_2}) = \overline{g}_{\alpha}^{\text{ZPE,H}}(x_H) - \frac{1}{2} g_{H_2}^{\text{ZPE}}(p^0_{H_2}) - \frac{1}{2}\Delta g_{H_2}^{\text{vibration}}(T, p^0_{H_2}) \tag{S11}$$

## S2. ZERO-POINT ENERGIES

The zero-point energies required to compute eq. (S10) are estimated with the `Phonopy` [S2, S3] software package. ZPE's are computed within supercells of $ZrMo_2$, $ZrV_2$, $ZrMo_2H_4$, and $ZrV_2H_4$. Low-energy hydrogen orderings were chosen with a stoichiometry of $x_H = \frac{4}{3}$ such that the hydrogen atoms are distributed over either the $A_2B_2$ or $AB_3$ sites for both Laves phase compounds. For both $ZrMo_2$ and $ZrV_2$, the difference in the ZPE's of hydrogen atoms distributed over either the $A_2B_2$ or $AB_3$ was $\approx 10$ meV per hydrogen atom. As the differences in ZPE's are relatively small, we use the same ZPE value for hydrogen atoms ordered over both tetrahedral interstitial sites. The ZPE for the hydrogen molecule is taken to be 0.27 eV [S4].

The calculation of ZPE in $ZrV_2$ presented additional complications. C15 $ZrV_2$ is predicted to be dynamically unstable by DFT as illustrated by the phonon dispersion curves of fig. S1. Experiments [S5–S7] on $ZrV_2$ have observed a low-temperature structural phase transition in this compound that is likely related to the unstable phonon modes of fig. S1. Anharmonic calculations are necessary to rigorously account for instability of C15 $ZrV_2$. However, we neglect these effects when computing the ZPE for hydrogen dissolved in this compound. The hydrides of $ZrV_2$ are predicted to be dynamically stable and did not display any imaginary frequencies.

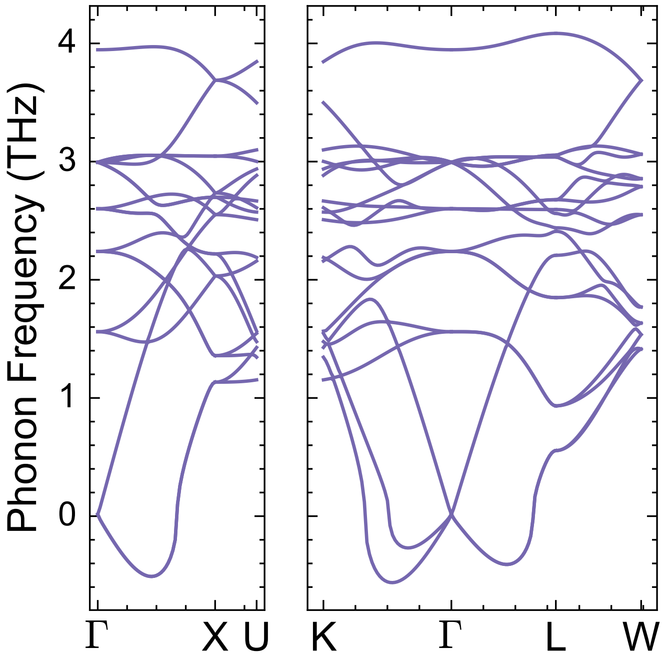


FIG. S1. Phonon dispersion curves of $ZrV_2$ computed within the harmonic approximation.

## S3. DFT COMPUTED VOLUME OF HYDROGEN-VACANCY ORDERINGS IN $ZrV_2$

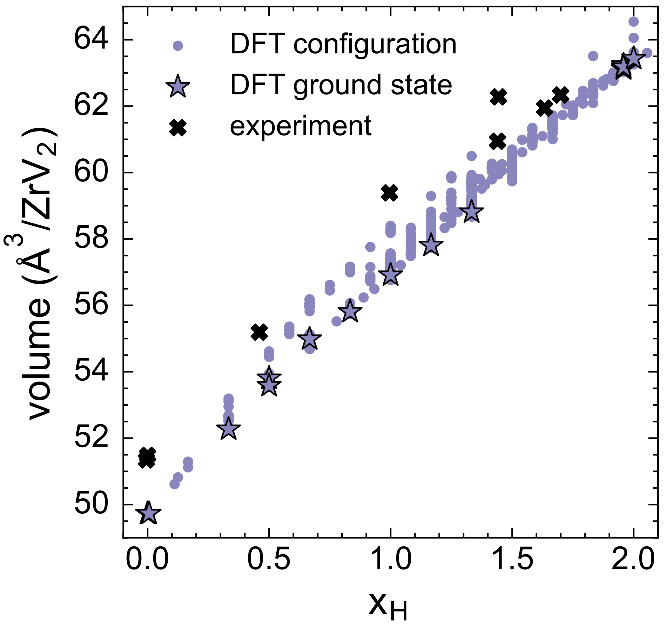


FIG. S2. Variation in the volume of hydrogen-vacancy orderings within $ZrV_2$ computed with DFT (light purple markers) compared against experiment (black crosses).

## S4. TRENDS IN THE HYDROGEN DISSOLUTION ENERGIES

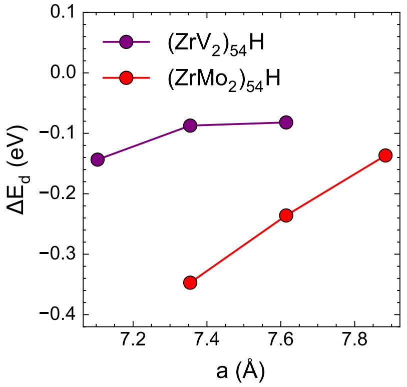


FIG. S3. Hydrogen dissolution energy difference ($\Delta$ $E_d$) between $A_2B_2$ and $AB_3$ sites in $ZrV_2$ and $ZrMo_2$ C15 Laves phases, computed in a 54-formula-unit $AB_2$ cell. The equilibrium lattice parameters are 7.35 Å for $ZrV_2$ and 7.61 Å for $ZrMo_2$.

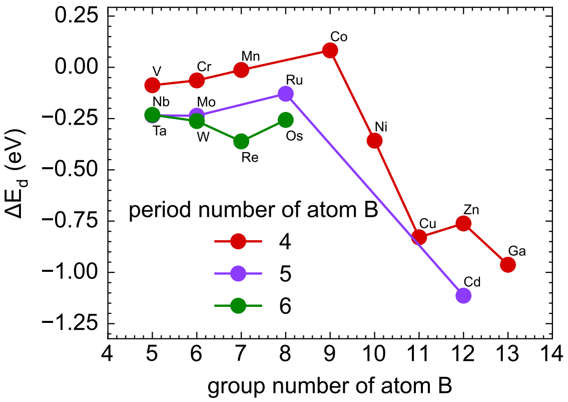


FIG. S4. Hydrogen dissolution energy difference ($\Delta$ $E_d$) between $A_2B_2$ and $AB_3$ sites, computed in a 54-formula-unit $AB_2$ cell of Zr-based Laves phases, with atom A = Zr and atom B varied across the periodic table.

## REFERENCES


[S1] M. Chase, *NIST-JANAF Thermochemical Tables, 4th Edition* (American Institute of Physics, -1, 1998).

[S2] A. Togo, First-principles Phonon Calculations with Phonopy and Phono3py, Journal of the Physical Society of Japan **92**, 10.7566/jpsj.92.012001 (2023).

[S3] A. Togo, L. Chaput, T. Tadano, and I. Tanaka, Implementation strategies in phonopy and phono3py, Journal of Physics: Condensed Matter **35**, 353001 (2023).

[S4] K. Miwa and A. Fukumoto, First-principles study on 3 d transition-metal dihydrides, Physical Review B **65**, 155114 (2002).

[S5] J. E. Doherty and D. F. Gibbons, A second order phenomenon in $ZrV_2$, physica status solidi (b) **44**, 10.1002/pssb.2220440148 (1971).

[S6] A. Lawson, More soft superconductors: ZrV2 and HfV2, Physics Letters A **36**, 8 (1971).

[S7] D. Moncton, Lattice transformation in the superconductivity ZrV2 by neutron diffraction, Solid State Communications **13**, 1779 (1973).
